# Non-coding sequence retrieval system for comparative genomic analysis of gene regulatory elements

**DOI:** 10.1186/1471-2105-8-94

**Published:** 2007-03-15

**Authors:** Sung Tae Doh, Yunyu Zhang, Matthew H Temple, Li Cai

**Affiliations:** 1 Biomedical Engineering Department, Rutgers University, 599 Taylor Road, Piscataway, NJ 08854, USA; 2 Dana-Farber Cancer Institute, 44 Binney Street, Boston, MA 02115, USA

## Abstract

**Background:**

Completion of the human genome sequence along with other species allows for greater understanding of the biochemical mechanisms and processes that govern healthy as well as diseased states. The large size of the genome sequences has made them difficult to study using traditional methods. There are many studies focusing on the protein coding sequences, however, not much is known about the function of non-coding regions of the genome. It has been demonstrated that parts of the non-coding region play a critical role as gene regulatory elements. Enhancers that regulate transcription processes have been found in intergenic regions. Furthermore, it is observed that regulatory elements found in non-coding regions are highly conserved across different species. However, the analysis of these regulatory elements is not as straightforward as it may first seem. The development of a centralized resource that allows for the quick and easy retrieval of non-coding sequences from multiple species and is capable of handing multi-gene queries is critical for the analysis of non-coding sequences. Here we describe the development of a web-based non-coding sequence retrieval system.

**Results:**

This paper presents a Non-Coding Sequences Retrieval System (NCSRS). The NCSRS is a web-based bioinformatics tool that performs fast and convenient retrieval of non-coding and coding sequences from multiple species related to a specific gene or set of genes. This tool has compiled resources from multiple sources into one easy to use and convenient web based interface. With no software installation necessary, the user needs only internet access to use this tool.

**Conclusion:**

The unique features of this tool will be very helpful for those studying gene regulatory elements that exist in non-coding regions. The web based application can be accessed on the internet at: .

## Background

While annotation efforts and gene prediction methods have begun the process of identifying protein-coding genes, robust high-throughput methods for detecting functional non-protein coding elements remain elusive [1]. Only about 2 percent of the human or mouse genomes consist of DNA sequences that are protein-coding regions [2,3]. The remaining vast majority of the genome consists of non-coding sequences (NCS). It has been shown that gene regulatory elements (GREs) reside in the NCS [4,5]. GREs have been broadly placed into two major functional groups: promoters and enhancers. Promoters are sequences that direct the precise locations of transcription start sites. Enhancers, repressor, and silencers, etc. are sequences that bind gene regulatory proteins and influence the transcription activity of a gene. GREs can be located upstream, downstream, or even internal to the target gene. GREs, therefore, act as switches to turn gene expression on or off and as modulators to increase or decrease expression. Traditionally, NCS have not received as much attention from investigators as protein coding sequences and GREs are generally poorly defined, mostly as only sequence motifs. Research is now focusing increasingly on non-coding sequences and specifically the search for NCS with regulatory functions. Identifying functional NCS and understanding their mechanism of operation will shed new insights into the understanding of the regulatory functions of transcription, DNA replication, chromosome pairing, and chromosome condensation [2,6]. In order for the full understanding and eventual control of biological function, not only must the genes involved in a particular function be identified but the regulatory elements that trigger and control the biochemical pathways that determine each gene's expression must also be well understood. However, searching for functional GREs within the NCS that comprise roughly 98% of the genome is not a simple task. The size and scope of this search brings with it many intellectual and experimental challenges that span computational biology and comparative functional genomics [7].

There are two commonly used methods for identification of functional GREs. The first uses gene expression analysis and the second uses comparative genomics. DNA microarray gene expression profiling is capable of evaluating thousands of genes across various experimental conditions. Bioinformatics approaches are used to cluster genes that show similar patterns of expression. Once genes with similar patterns of expression are identified, they are searched within their upstream sequences to identify over-represented or conserved sequence motifs [8,9]. Sequence alignment algorithms employed by the comparative genomic methods are powerful in identifying conserved sequences in non-coding regions located in and around genes with the same function, known as homologous genes, from diverse species. Homologous genes usually have the same function and may also have similar regulatory elements that control this function. Functional regions (which consist of protein coding regions along with regulatory regions) experience selective pressure against change and therefore have a higher level of sequence conservation across a wide range of species than non-functional regions. Ideally, selective pressures would allow for non-functional sequences to diverge due to evolutionary drift while leaving functional regions with high similarity [1,10-17]. DNA sequence comparison of the human and mouse orthologous genes have indicated that conserved NCS are enriched significantly in regulatory sequence regions [4,18,19]. Subsequent to the identification of putative regulatory elements by sequence comparison, the confirmation of biological function will depend upon experimental assays. Transcriptional regulatory regions in genes from humans, mouse, Fugu fish, *Caenorhabditis elegans*, *Drosophila*, and yeast [5,6,20-24] have been identified. The power of comparative genomics analysis is enhanced significantly when genomic sequences are available from a number of related species that have diverged sufficiently. This reduces the chances of conservation among non-functional elements. By comparing multiple genomes, it can help to determine which conserved elements are more likely to be functional [5,21,25,26].

Whether the focus is on genes with similar expression patterns or those expected to have the same function, both analysis methods require the retrieval of NCS for the identification of functional sequence elements. Currently, the process of retrieving these sequences is performed manually from a wide range of sources. No efficient method is available for retrieving NCS quickly and systematically at a single source. To facilitate the analysis of NCS for functional regulatory elements, we present here a web-based non-coding sequences retrieval system (NCSRS) that performs the automated retrieval of non-coding sequences among genomes of different species. A previously developed application for retrieving NCS, called Retrieval of Regulative Regions (RRE) [27] parses annotation and homology data from NCBI to identify NCS. This parser requires local installation but also requires a local copy of desired genomes and annotation files. A web based application is also available but only a few genomes are currently available and RRE utilizes annotation data from only NCBI. The NCSRS requires no installation or local management of genome sequence databases and utilizes annotation information from both NCBI and Ensembl. Currently, NCSRS has 15 genomes (containing over 85 Gigabyte of DNA sequence data) with sufficient annotations available for NCS retrieval.

## Implementation

### Annotation and sequence information

There are two major groups currently working on genome annotation. Data from these two sources serve as the source of the annotation information that comprises the core for this tool. These include NCBI RefSeq [28] and Ensembl [29]. RefSeq is a partially manually curated annotation database which includes information based on predicted mRNAs and proteins. The genes with manually curated mRNA information are labelled with the "NM" prefix while the genes based on predicted mRNA information are labelled with an "XM" prefix. Ensembl's gene predictions are automated but all predictions are based on experimental mRNA evidence. The Ensembl annotation system marks predicted genes as either known or novel based on the level of experimental support and information from other annotation sources. The number of genes annotated by Ensembl and Refseq are listed in table 1 and 2. Table 3 shows the number of genes annotated in Refseq are also included in the Ensembl system along with the percentage of overlap between the two with respect to the total number of annotated genes in the Ensembl system. The pros and cons of each will not be discussed here but rather it is emphasized that this tool offers users the freedom to choose.

**Table 1:** Statistics of gene annotation for ENSEMBL and NCBI.

**ENSEMBL – **as of 12/06/06						
**Organism**	**Assembly**	**Genebuild Date**	**Version**	**Known**	**Novel**	**Total Predictions**
***Human***	NCBI 36	Aug 2006	41.36c	22205	1019	69185
***Mouse***	NCBI m36	Apr 2006	41.36b	21839	2599	71259
***Chicken***	WASHUC 1	Dec 2005	41.1p	5123	5417	76146

						
**NCBI – **taxonomy browser and Unigene as of 12/06/06						

**Organism**	**Assembly**	**GenBank Date**	**UniGene Build**	**Entrez Genes**		**Total Unigene Clusters**
***human***	NCBI 36	Oct 2006	197	38597		85590
***mouse***	NCBI m36	Oct 2006	159	60745		64618
***chicken***	WASHUC 1	Aug 2006	31	24313		30837

Known – genes that have species-specific protein sequences already available in the public sequence databases. Novel – genes that could not be mapped with confidence to existing entries. Total Predictions – the number of 'known', 'novel' and 'pseudogenes' predicted by the Ensembl analysis and annotation pipeline.

Entrez Genes – number of genes defined by sequence and/or located in the NCBI Map Viewer. Total Unigene Clusters – the number of non-redundant sets of gene-oriented clusters automatically partitioned by UniGene.

**Table 2:** Statistics of homology prediction for human, mouse, and chicken

**Ensembl (mart 41)**		**Baseline Species for Homology Search**		
		
		***Human***	***Mouse***	***Chicken***
**Species of Homologous Genes**	***Human***	-	13049/46.7%	9839/50.7%
	***Mouse***	12036/38.6%	-	11698/60.3%
	***Chicken***	11773/37.7%	12187/43.6%	-
**Total number of genes**		31206	27964	19399

**Homologene (release 53)**		**Baseline Species for Homology Search**		
		
		***Human***	***Mouse***	***Chicken***

**Species of Homologous Genes**	***Human***	-	16325/73.0%	10498/84.0%
	***Mouse***	16325/41.2%	-	10299/83.3%
	***Chicken***	10498/26.5%	10299/46.6%	-
**Total number of genes**		39605	22364	12500

The homolog data table files for each of the baseline species were queried to find the total number of genes along with the number of homologous genes that are present in another given species' genome. Similarly the homologene.data file was used to generate the homologene statistics. Shown are the number of homologs and the percentage of coverage (the number of genes that have homologs in a particular species' genome divided by the total number of genes for the baseline species.)

**Table 3:** Analysis of known and predicted genes for chicken, rat, mouse, and human from Ensembl Mart v.41

**Species**	**NM (known)**			**XM (predicted)**		
	
	**Refseq known to Ensembl**	**Ensembl**	**Percentage**	**Refseq known to Ensembl**	**Ensembl**	**Percentage**
***Chicken***	2726	24939	10.93%	1	24910	0.00%
***Rat***	9119	37825	24.11%	9731	38778	25.09%
***Mouse***	21336	36898	57.82%	16931	46566	36.36%
***Human***	29836	62076	48.06%	9849	63575	15.49%

						
			
	**TOTAL**					
				
**Species**	**Refseq known to Ensembl**	**Ensembl**	**Percentage**			
			
***Chicken***	2727	49849	5.47%			
***Rat***	18850	76603	24.61%			
***Mouse***	38267	83464	45.85%			
***Human***	39685	125651	31.58%			

The RefSeq annotation data (RefGene.txt, RefLink.txt) is obtained for each genome from the Human Genome Browser at UCSC [30] ftp site[31] as are some of the genome sequences. The remaining genome sequences along with the Ensembl data (gene, transcript, and structure files for each genome) are obtained from Ensembl's ftp site[32]. Both sets of gene annotations are downloaded and then processed to serve as the basis for building a genome map that contains the location of each gene and all its exons. Ensembl includes 5' and 3' UTR's along with coding exons when counting the total number of exons. 5' and 3' UTR's are also listed on their website as exons and are included in the downloadable annotation files as exons. Therefore, following this convention, 5' and 3' UTR's have also been included in the list of exons by NCSRS. In those cases where multiple transcripts are available, the transcript with the greatest number of exons is used. Because not all the transcript information is used to define sequences in the intron region there is the potential that an exon from an unused transcript variant may not be shared with the utilized transcript. In this case, the unshared exon would be "hidden" from the annotation and returned as part of the non-coding sequence. However this is not relevant in the majority of cases as "hidden" exons are not the norm. Furthermore, this is limited to only the intragenic region and would not affect the up and down stream sequences.

In this paper non-coding regions refer to all sequences that are not exons. The definition of exons will follow the convention used by Ensembl of defining exons as the set of 5' UTRs, coding exons, and 3' UTRs. Ensembl includes 5' and 3' UTRs in their total exon counts both on their website and in the downloadable annotation files [29,33]. While it is arguable that UTR's can be considered non-coding regions, for the sake of consistency and clarity, the convention followed by Ensembl is maintained.

### Homology prediction

The RefSeq annotation information, called Homologene [34], is the basis for the homologous gene prediction system. This system is implemented in the NCSRS by using the single "homologene.data" file [35] for gene annotation information from RefSeq. This file is a list of the sets of homologous genes for all genes annotated by RefSeq. Ensembl has its own homology prediction method and its output is organized in a set of multiple files. The Ensembl annotation information is the basis for its homologous gene prediction and therefore when using Ensembl's annotation information, Ensembl's homology prediction results are used. Simple analysis of the data generated by the two homology prediction methods shows that both systems have good coverage for those genomes most commonly used for research (see Table 2). As the annotations improve, the results of the predictions will also improve in accuracy and percent coverage.

### Input

**Figure 1:**
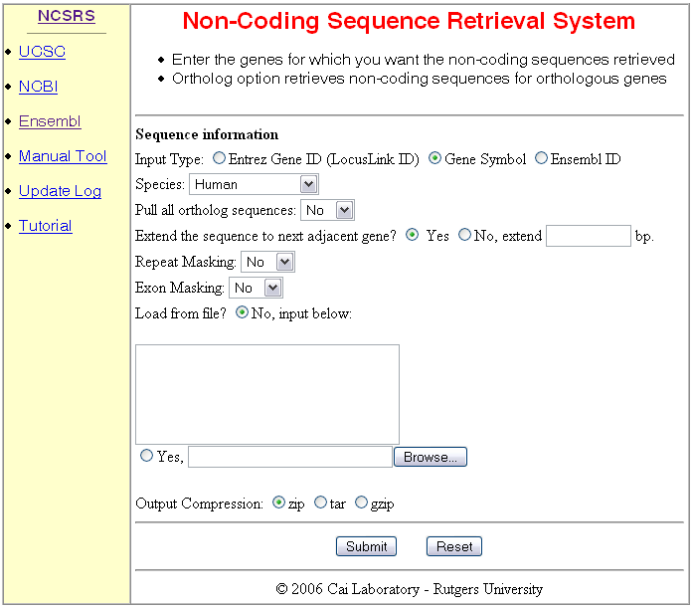
Snapshot of the web based user interface for the NCSRS. The user interface allows the user to input the HUGO (Human Genome Organization) ID, i.e., Entrez gene ID (LocusLink ID), Gene Symbol, and Ensembl ID numbers and set the other search options.

**Figure 2:**
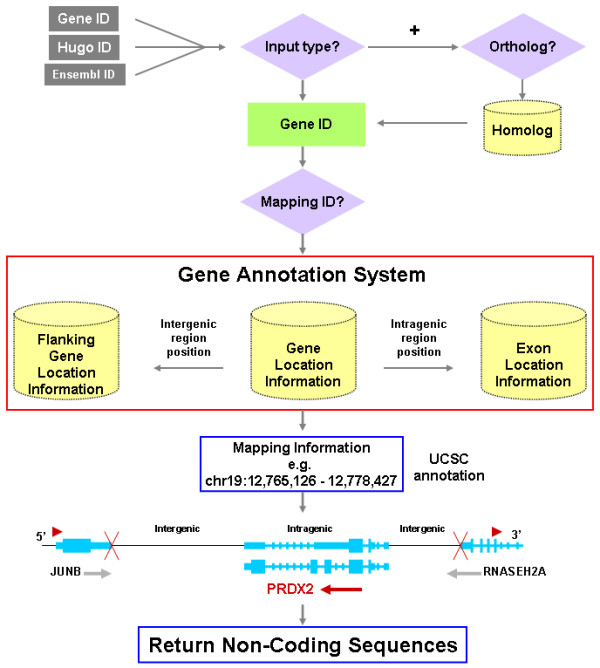
Work flow diagram of the NCSRS. The Refseq annotation uses Entrez gene IDs as the database key while Ensembl uses gene stable IDs. The input ID is converted into the appropriate database key if necessary. Entrez gene IDs are used directly for the Refseq annotation but are converted to gene stable IDs for the Ensembl annotation. Gene symbols are translated into Entrez gene IDs and gene stable IDs. Once the database keys are acquired, the homologous genes can be identified using the available homology databases if the "pull ortholog" option is activated. The database key is then used to access the mapping information that has been compiled from the annotation data. The mapping information is then used to locate the relevant sequences. These sequences are extracted then copied to a new ".fa" file with FASTA sequence format; and the annotation information about the exons is written to the ".exon" file. Thus, for each requested gene, there are one pair of files for each genome.

The user's input of the desired search options on the user interface (Figure 1) determines the work-flow path (Figure 2). The search options include the input type, genome or genomes from which sequences will be extracted, range of sequence extension, exon masking, and output format. The user inputs the individual gene or set of genes of interest. The input can be either the Entrez gene id or HUGO gene symbol (default) but must be the same for all the genes of a given search. The non-coding sequences from a specific species can be returned by selecting the desired species from a pull down menu or from all species with a known orthologous gene, by activating the "pull all ortholog sequences" option according to the homologene database.

### Mapping

Using the annotation information all annotated genes are sorted based on chromosomal position. Then the start and stop locations for all coding regions are identified. By identifying the coding regions we can also determine the locations of the non-coding regions using genomic position information, or mapping, of a specified gene and its flanking genes. The non-coding sequences are identified simply as those located between the adjacent identified exons. The information for each gene's non-coding region is then written to a new set of files that are used by the NCSRS. This non-coding region annotation serves as the basis for the locations of the end points for each intergenic and intragenic region.

### Retrieval of non-coding sequences

**Figure 3:**
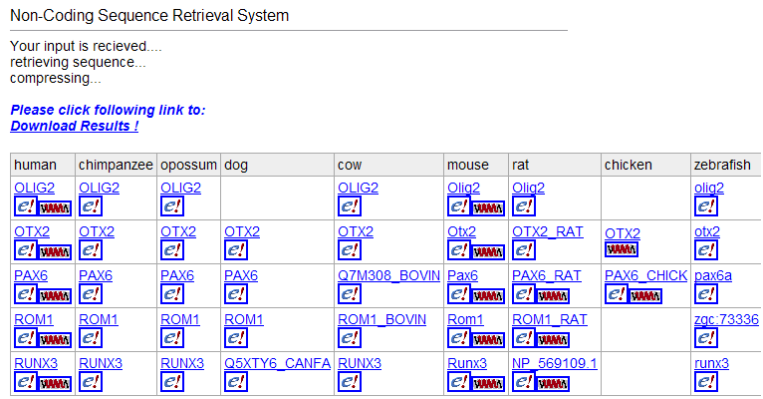
An example webpage that display the results for the NCSRS. The sequences and annotation information written to the FA and EXON files respectively are bundled and zipped into a single file that can be accessed by the "Download Results!" link. A table with links to NCBI, UCSC genome browser and Ensembl for the gene and specific species is also provided.

Using the locations of non-coding regions the appropriate genomic sequence is identified. The genome sequence file is read and the specified sequences are extracted and copied to a new file according to the appropriate position information. The NCSRS by default outputs the non-coding sequence of the specified gene starting from the end of its adjacent gene and ending with the start of the other adjacent gene (as marked with 2 "X"s in Figure 1). Unless specified otherwise the NCSRS also outputs the intergenic sequences, exons and introns. The boundaries for the flanking regions can be arbitrarily set by specifying the extension length in the options, e.g., with a specified extension length of 5000 bp. Then, 5000 bp up- and down-stream of the queried gene will be included in the extraction irrespective of the location of neighbouring genes. Each sequence has a pair of associated files: FASTA format sequence file with file extension ".fa" and EXON definition file with file extension ".exon", both with file names generated using the UCSC annotation format. The ".fa" file contains the sequence in FASTA format. The first line of the ".exon" file defines the span of the coding region. The first line also contains other information such as the name of the gene the chromosome the gene is located and the chromosomal position of the extracted NCS. The following lines list pairs of locations which represent the start and end of all the exons. These locations are relative to the start of the sequence in the ".fa" file. The set of sequences and exon annotation files are packaged as a compressed file that is available for download through a link on the results webpage (Figure 3). The result webpage also has a table of the gene or genes returned, for each genome that is currently available, along with a link to the results for each genes query of NCBI's entrez gene site [36], a gene map view from UCSC's genome bioinformatics website [37], and further gene information at Ensembl's website [38].

### Updating

Both annotation systems, Refseq and Ensembl, are works in progress and   with subsequent releases, the annotations will improve in scope and accuracy. New genome assemblies will also continue to be released for new and existing genomes. As the sequence and annotation information which serve as the basis for this system are refined, the sequences generated by this system will improve. Therefore, it is critical that the genomes and annotation information are kept up to date. The NCSRS will be updated automatically on a weekly basis to ensure that the most recent information is always available.

### Hardware and software

The NCSRS uses a single computer that acts as both the server and database. There is also a developmental computer which is used for updating, designing new applications, and troubleshooting. The main server uses Dual Intel^® ^Xeon^® ^Processors at 3.0 GHz, with 4 Gb RAM and 500 GB Hard Disk space and runs apache 2.2 as its web server. The scripts and programs used by NCSRS for building and accessing the databases are written predominantly in PHP and Perl.

## Results

We have developed a web-based sequence retrieval system that quickly and easily extracts non-coding sequences associated with a specific user defined gene set from a single and/or multiple genomes. The NCSRS efficiently delivers non-coding sequences for specified genes or gene sets using a user-friendly interface from a single site. This system eliminates the need to manually sift though genome sequences and look for annotation information from multiple sources. This will help eliminate human errors as well as increase throughput for those investigating gene regulatory elements. The system also allows the user to specify the gene or set of genes for retrieval while maintaining a simple user interface, enabling the user to apply their expert knowledge without having to spend a lot of time learning how to use the system. Another option that is important for those seeking to elucidate functional NCS is masking. Repetitive sequence elements found in the genome can cause sequence alignment algorithms to predict conserved elements that do not have gene regulatory function. For this reason, there is an available option to mask sequences as repeated sequences to allow for alignment algorithms to ignore repeated sequences [1].

This system has great flexibility in its potential applications. An important and unique feature is that if the user intends to apply this tool to a comparative genomics approach, the user can obtain the sequences for multiple species by simply selecting the "pull all orthologs" option. Once the sequences are returned, a multiple sequence analysis can be performed for each set of homologous gene sequences. The system's ability to return sequences from multiple genomes in one run greatly increases the efficiency and speed of the system. Furthermore, it has been shown that increasing the number of genomes used in alignment analysis increases the   signal-to-noise ratio and, if specific genomes are selected carefully,   increases the likelihood of correctly predicting functionality [1]. If microarray data is the basis for analysis, the system's ability to handle multiple genes in a single query allows for the user to input multiple genes with similar expression patterns at one time to return the desired sequences. It is even possible to combine the two approaches and obtain sequences for all homologous genes of a set of genes with similar expression profiles. This allows for the system to be utilized by those who seek to learn more about genome-wide networks through their analysis [39].

## Conclusion

NCSRS combines a number of available genomic resources (a total of over   85 Gb sequences) and applies them to the specific task of identifying and retrieving non-coding sequences in an up to date web based application that is easy to use and requires no maintenance by the user. The unique features of this tool will be very helpful for those studying gene regulatory elements that exist in non-coding regions. Future work will include incorporating NCSRS with a program that analyzes non-coding sequences using a multi-sequence alignment algorithm and identifies highly conserved regions [10-15]. This pipeline will be designed to be able to rank potential gene regulatory elements according to the likelihood of functionality using sequence motif information of known transcription binding factors [40]. Ultimately the seamless integration of these two tools with the NCSRS will be implemented into a gene regulatory element finder pipeline. This will allow future experimental work and resources to focus on the verification of potential regulatory elements to those conserved elements that have a theoretically basis for regulatory function and therefore increase overall efficiency. The proposed pipeline will serve as the initial selection process for targeting experimental verification.

## Availability and requirements

Project name: NCSRS

Project home page: 

Operating system(s): Platform independent

Programming language: Perl, PHP

Licence: GPL

Any restrictions to use by non-academics: None

## Authors' contributions

LC identified the need to develop such a system, initiated the project, and designed the basic functions. SD and YZ wrote the source code for the software and web interface and contributed with ideas on overall design, feature requirements, and implementation. All authors participated in the drafting of the manuscript and approved the final version.
